# Toxic effects of 70% ethanol extract of *Moringa stenopetala* leaf (Baker f.) Cufod. (Moringaceae) on fetus and placenta of pregnant Wistar rats

**DOI:** 10.1186/s12906-023-03937-6

**Published:** 2023-04-03

**Authors:** Hussen Abdu, Wondwosen Ergete, Ashenif Tadele, Samuel Woldekidan, Abiy Abebe, Girma Seyoum

**Affiliations:** 1 Department of Anatomy, School of Medicine, College of Health Sciences, Addis Ababa University, P.O. Box 1176, Addis Ababa, Ethiopia; 2 Department of Pathology, School of Medicine, College of Health Sciences, Addis Ababa University, Addis Ababa, Ethiopia; 3 Traditional and Modern Medicine Research Directorate, Ethiopian Public Health Institute, Addis Ababa, Ethiopia

**Keywords:** Fetus, Developmental retardation, *Moringa stenopetala*, *Placenta*, Rat, Toxic effect

## Abstract

**Background:**

*Moringa stenopetala* leaves (Baker f.) Cufod. (Moringaceae) are used as a staple food and traditional medicine for treating various diseases like malaria, hypertension, stomach pain, diabetes, elevated cholesterol, and removing the retained placenta. Its prenatal toxicity study is minimal. Thus, this study aimed to assess the toxic effects of a 70% ethanol extract of *Moringa stenopetala* leaf on the fetuses and placentas of pregnant Wistar rats.

**Method:**

Fresh leaves of *Moringa stenopetala* were collected, dried at room temperature, ground to powder, and extracted using 70% ethanol. For this study, five groups of animals, each containing ten pregnant rats, were used. Groups I–III were experimental groups and treated with 250, 500, and 1000 mg/kg body weight of *Moringa stenopetala* leaf extract, respectively. Groups IV and V were pair-fed and *ad* *libitum* control groups. The extract was given during gestation days 6 to 12. The fetuses were recovered at day 20 of gestation and examined for the presence of developmental delays, gross external malformations, skeletal and visceral defects. Gross and histopathological changes in the placenta were also evaluated.

**Results:**

Compared to the pair-fed control group, maternal daily food intake and weight gain were reduced in the 1000 mg/kg-treated group during the treatment and post-treatment periods. A significantly higher number of fetal resorptions was also seen in the 1000 mg/kg treatment group. The crown-rump length and fetal and placental weights were all significantly reduced in pregnant rats given 1000 mg/kg. However, there were no visible malformations in the visceral organs as well as external genitalia in all the treatment and control groups. About 40.7% of the fetuses in the 1000 mg/kg treated rats had no proximal hindlimb phalanges. In addition, light microscopic investigations of the placenta in the high-dose treated rats revealed structural changes in the decidual basalis, trophoblastic zone, and labyrinthine zones.

**Conclusion:**

In conclusion, consumption of *M. stenopetala* leaves at a higher dose may have toxic effects on the development of rat fetuses. At a higher dose, the plant extract increased the number of fetal resorptions, reduced the number of fetuses, decreased the fetal and placental weights, and alter the placental histopathology. Thus, it is recommended to limit the excess feeding of *M. stenopetala* leaves during gestation.

## Introduction

Herbal medicine is one of the most traditional forms of treatment for diverse diseases. It has enjoyed a relatively extraordinary contribution for apparent reasons like its cost-effectiveness, availability, and blend with the socio-cultural life of the people [1]. In developing countries, the contribution of herbal medicine is as much as 80% [2].

Many ethnobotanical and phytochemical investigations on medicinal plants have been carried out to look into safer immunomodulator medications [3]. The secondary metabolites found in medicinal plants, such as terpenoids, phenolics, saponins, flavonoids, and alkaloids, may have therapeutic use in the development of new drugs [4].

Although xenobiotic agents, or substances alien to the human body, are present in the medicinal plants used to make herbal medicines, it should be emphasized that the biotransformation products of these processes may be poisonous. In addition to the acute effects, which are easily linked to their intake, long-term consequences can be asymptomatic but result in a serious clinical picture that may even be lethal [5]. Both internal and external factors, including phagocytes, cytochrome P450 mono-oxygenase (CYP), irradiation, and exogenous substances, can cause the production of ROS. Nevertheless, ROS can potentially cause damage by forming irreversible or covalent bonds with biological macromolecules [6]. This could lead to birth abnormalities, growth retardation, and, in extreme circumstances, in utero death during the prenatal period [7, 8]. Because of its poor antioxidant defence, the developing embryo is particularly vulnerable to high amounts of ROS, especially in the initial phases of organogenesis [9]. Despite the fact that placental enzymes aid in the fetus’ defence against oxidative stress [10].

The embryo is more susceptible to oxidative stress, and teratogens that can alter redox statuses, including thalidomide, phenytoin, and ethanol, will damage fetal development [7]. Oxidative stress leads to lipid peroxidation, reactive oxygen species production, DNA fragmentation and cell apoptosis [11]. In light of this, it has been hypothesized that oxidative stress contributes to the pathogenesis of a number of birth defects, such as skeletal abnormalities, limb abnormalities, and neural tube defects [12–14]. Fortunately, antioxidants can prevent these consequences by altering gene expression, transcription factor signalling, and cell cycle alterations [7].

In Ethiopia, over 70% of the people be contingent on traditional medicines for their primary healthcare needs, and more than 95% of the preparations are made from plant sources [15, 16]. Beyond their availability and importance, expressly in Africa, the shared problems of herbal medicine are their scarce quality control and safety issues [17].

*Moringa stenopetala (M. stenopetala),* is a fast-growing, softwood, multipurpose plant, native to north-east tropical Africa and India. It is belonging to the family Moringaceae and is well-recognized for its nutritional and medicinal values [18–20]. It is often named an African Moringa tree because it is widely cultivated in southern Ethiopia, North Kenya, and Eastern Somalia [21, 22]. In Ethiopia, this plant is identified by various vernacular names. It is frequently named Shiferaw in Amharic [23], Halako in Gofa and Wolyita, Shelagta in Konso, and Haleko in Derashe [24–27]. All parts (root, bark, gum, leaf, fruit, flower, seed, and seed oil) of the plant possess both nutritional and medicinal values [27–29]. The leaves are consumed as an indigenous vegetable food and used as the TMs for treatments of malarial, hypertension, stomach pain, diabetes, high cholesterol, expel of a retained placenta [24–27]. It is additionally utilized for treating a cold, fever [28] cancer [29] visceral leishmaniasis [30] leprosy, and cough [31]. The flowers are good sources of nectar for honey, and the seeds are operated to purify turbid water [24]. The barks are managed to soothe a cough, and the grinded wet or dried roots are used to treat malaria and strenuous labour [31, 32].

Phytochemical studies have found alkaloids, saponins, tannins, steroids, phenolic acids, glucosinolates, flavonoids, and terpenes [33]. Moreover, *M. stenopetala* leaves are rich in vitamin B, potassium, calcium, and protein, as well as -carotene and other secondary metabolites, antioxidant vitamins (A, C, and E), and vital micronutrients (selenium/zinc) [34, 35]. These active ingredients are liable for their wide-ranging pharmacologic actions. Moringa leaves can also be a good source of antioxidant molecules like glutathione, catalase, peroxidase, and vitamins that can slow down or stop the oxidation of other molecules, so shielding cells from damage brought on by exposure to free radicals, particularly reactive oxygen species. Furthermore, phenolic substances with potent antiradical action are present in the plant [36, 37].

Yet, this plant contains poisonous elements that might affect the host system. Alkaloids are important secondary metabolites present in the plant with a high medicinal potential, but there are claims that ingesting natural plant alkaloids during pregnancy may have various unintended adverse effects on both mother and fetuses. This is known as the “teratogenic impact” of natural alkaloids and is caused by a disturbance in the cholinergic neurotransmission, which results in developmental defects in the fetuses [38–40]. Alkaloids and terpenoids have also been linked to increased levels of bone morphogenetic protein-7 (BMP7), a kind of transforming growth factor (TGF-), also known as osteogenic protein-1, in the decidua of the placenta, which is close to the site of implantation [41].

Any medication or substance used by pregnant women, whether natural or synthetic, must always be weighed against the risks and benefits [42, 43]. The embryo or fetus may develop abnormally as a result of prenatal chemical exposure. This may show up as the existence of deformed organs, developmental delays, decreased functionality, full or partial organ agenesis, and fetal mortality [44]. Independent of their toxicity to the mother, chemicals can directly impair fetal growth while pregnant. Many of these substances can cross the placental membrane after maternal exposure to them and reach the embryo or fetus. The typical development of their progeny could be hampered by these substances [45].

Currently, there are conflicting reports on the safety of different parts of *M. stenopetala*. For example, ethanol extract from the leaves and seeds of this plant contains toxic substances either extracted by organic solvents or formed during the process of extraction [46]. Besides these safety issues, the plant is, however, used as a food source and traditional medicine [47]. In addition, prenatal toxicity study, which is the third-ranking issue next to cardiovascular and hepatic toxicities [48], is compulsory before drug preparations [49].

Several acute, sub-chronic, and chronic toxicological investigations have been done and published on different parts of the plant. However, investigations on its fetal toxicity study are minimal. In addition, in the previous investigation on the teratogenic profiles of the leaves of *M. stenopetala* on rat embryos and fetuses [50], the number of animals used was few and many parameters like gross external and visceral organ examinations and skeletal assessments were not addressed. Therefore, the current study aimed to investigate the toxic effects of a 70% ethanol extract of *M. stenopetala* leaf on 20 days old fetuses and placentas of pregnant rats. In this study, indications for fetal growth, histopathology of the placenta, and developmental statuses of the visceral organ, as well as the skeletons of the rat fetuses were assessed. In addition, the data generated in this study could also be used as a baseline for further comprehensive developmental toxicity studies.

## Materials and methods

The protocol employed in this investigation conformed with pertinent institutional, national, and international laws and regulations governing studies involving both plants and animals. Furthermore, we employed essentially identical experimental procedures and protocols to those used in earlier research by Abebe et al. [49] and Fentahun et al. [51] and we mostly used the same terminology in our explanations.

### Setting of the study and experiment

The study was conducted in the laboratory of the Ethiopian Public Health Institute (EPHI), Modern and traditional medicine, and Anatomy and Pathology Departments of Addis Ababa University (AAU) from December 2018 to September 2021. The study assessed the developmental toxicity of *M. stenopetala* leaf extract in rat fetuses following administration of the extract to pregnant rats during the critical period of gestation in which organogenesis takes place.

### Collection of plant material and preparation of extract

Fresh leaves of *M. stenopetala* were collected around Arbaminch city, located in the southern parts of Ethiopia which is 500 km far from Addis Ababa. There was no specific official authorization needed to harvest the leaves of the plant because it grows wildly in southwest Ethiopia, and it is not an endangered species. Authentication of the plant was carried out by Dr. Asfaw Debela a senior researcher and taxonomist in the EPHI, where a voucher number AL-001 was given and deposited in the herbarium for future use [52]. The leaves were cleaned, washed, mangled, sliced into smaller pieces, dried under shade, and then ground to powder using an electric mill and stored at room temperature. The powder was mixed with 70% ethanol (EtOH) in the ratio of 1:10 powder to solvent in Erlenmeyer flasks wrapped with aluminium foil and then rotated for 24 h using an orbital shaker at 100 revolutions per minute (RPM). It was then filtered using Whatman No, 1 filter paper (18 cm in diameter). The solvent was removed from the filtrate by using a rotatory evaporator (BÜchi Rota Vapor R-205, Switzerland) at 40 °C and 175 millibar pressure. The crude extract was dried by placing the filtrate into a hot water bath at 45 °C. The dried crude extract was then placed and packed in a wrapped glass container and stored in a refrigerator at − 20 °C until used for the experiment [53].

### Experimental animals

All protocols were approved by the Institutional Review Board (IRB) of the College of Health Sciences, Addis Ababa University (Form AAUMF 03–008, Elements Reviewed AAUMF 01–008). The animals were handled in compliance with the International Standards for the Handling of Experimental Animals [54] and ARRIVE guidelines and following OECD Test Guideline 414 for the care and use of laboratory animals during prenatal developmental toxicity studies [55].

In the current study, healthy, nulliparous Wistar albino rats, weighing 225–240 g, and ages 10–12 weeks were used. The animals were obtained from the EPHI animal breeding unit. They were kept in the animal house of the Traditional and Modern Medicine Research Directorate (TMMRD) of the EPHI and acclimatized to the environment for 1 week before the actual experiment. The animals were placed in stainless-steel cages in an environmentally controlled room with temperature (23 ± 3 °C), relative humidity (50% ± 10%), and 12-h light and dark cycles. During the adaptation period, all animals were fed a standard pellet (composed of carbohydrate (75%), protein (16%), fat (55%), calcium (3.6%), and phosphorus (0.4%) with free access to tap water ad libitum*.*

After 1 week of adaptation, one randomly selected male rat with proven fertility was placed into a cage containing one nulliparous female rat. After an overnight mating, female rats were inspected for the presence of a copulatory plug the next morning and a vaginal smear was taken for microscopic determination of the presence of sperm cells. The pregnancy was confirmed after checking spermatozoa in the vaginal smear test. The date of sperm detection within the vaginal smear was considered as day- one of the pregnancy [56].

### Experimental design

The current experiment was designed to assess the in-vivo toxic effect of 70% ethanol extract of *M. stenopetala* leaf on 20 days old fetuses and placentas of pregnant Wistar rats. It investigated the developmental toxicity of 70% ethanol extract of *M. Stenopetala* leaf in near-term fetuses and histopathological changes of the placenta. Fifty pregnant rats with a unique number marked on the tail of each rat using a permanent marker were randomly assigned into five groups. The first three (groups I-III) were treatment groups and they were treated with *M. stenopetala* leaf extract orally at doses of 250, 500, and 1000 mg/kg body weight, respectively. The doses were selected based on a previous efficacy study [57]. The fourth group (IV) was the pair-fed control group that received distilled water at a volume of 2 ml/100 g body weight and was fed with a volume that was matched to the amount consumed by the experimental groups. The last group was an ad libitum control that was fed freely and remained untouched throughout the experiment. The treatment period was from day-6 through day-12 of gestation. The daily food intake of each animal was recorded every morning, and animals were weighed and weight gain was recorded on days 1, 6, 12, and 20 of gestation. During the experiment, the pregnant rats were inspected daily for any behavioural changes and signs of noxiousness. The observations in the experiment were dimly completed by the investigator unaware of the treated and control rats [58, 59].

At the end of the experiment, on gestation day 20, the animals were euthanized by intraperitoneal injection of pentobarbital sodium (150 mg/kg body weight) [60]. The pregnancy outcomes, developmental parameters, like morphological scores, ossification centers, gross morphological and visceral organs, and histopathology of the placenta were examined.

#### Evaluation of pregnancy outcomes

The pregnancy outcomes were evaluated by counting the number of implantation sites, resorptions sites, live fetuses, dead fetuses, and sex of the fetuses. To evaluate these parameters, the anterior abdomen of the pregnant rats was opened, and the uterine horns were exposed, removed, and examined. The number of implantation sites was checked by counting the metrial glands which are yellowish nodules located along the mesometrial border of the uterine horns. The metrial nodules unoccupied by living or recently dead fetuses represented the number of earlier resorptions. The number of live or dead fetuses was determined by exerting gentle pressure on them. The removed uterine horns were placed in a clean glass container and then incised along the anti-mesometrial border of each horn. Instantly, each fetus was revealed and detached from the placenta and all fetal membranes were weighed. The placental weight was recorded. The sex of the fetuses was identified, and the weight and CRL of each fetus were measured. The procedures followed the methods described by Seyoum and Persaud that were adopted for the in vivo toxicological study from Brown and Fabro [61, 62].

#### Morphological evaluation

Once each fetus was revealed and separated from the corresponding placenta, all fetuses were examined from head to tail for the presence of any gross developmental malformations. The assessment includes craniofacial anomalies (exencephaly, anencephaly, microphthalmia and anophthalmia); limb abnormalities (syndactyly, adactyly, polydactyly); vertebral column anomalies (neural tube defect, kyphosis, scoliosis), a disorder of tail development (missing tail); and external genitalia malformations. Two to three fetuses per litter were randomly selected for examining skeletal developmental delays. The rest of the fetuses were fixed in Bouin’s solution for 2 weeks (aqueous saturated solution of picric acid 75%, formalin 25%, and glacial acetic acid 5%) for visceral examination [61, 62].

#### Visceral examination

Following gross external examination of the fetuses, visceral organs were examined by serial sectioning that was made on the body of fetuses fixed in Bouin’s solution for 2 weeks. The sectioning was performed by a surgical blade, based on the Modified Wilson technique [63, 64]. The sections were done craniocaudal at intervals of 1–2 mm with the help of dissecting microscope (XTL3101, 6× magnification). The sectioning was started at the jaw and passed dorsally superior to the ear. The tongue was removed, and the palate was examined for the presence of any cleft. In addition, a coronal section on the head and a transverse section on the neck and parts below were sequentially done. The following organs were assessed for any visible anomalies: brain (hydrocephalus, dilation of ventricles, microphthalmia/anophthalmia) craniofacial region (nasal septum defect, cleft palate), thoracic region (Lungs: lobar defect, heart: septal defect, retro-oesophageal aortic arch), abdominal region (liver, stomach, and gut anomalies), and pelvic region (Kidneys: agenesis, ectopic kidney, and hydronephrosis, gonads: testes and ovarian anomalies) [49].

#### Skeletal staining and evaluation

Skeletal staining was done by employing the method of Dawson [65–67]. Depending on the litter size, 2–3 fetuses per litter were randomly selected and killed by an overdose of pentobarbital. These fetuses were eviscerated, and all internal organs were taken out through a midline incision on the anterior abdominal wall. The eviscerated fetuses were then placed in a small bottle containing 95% ethanol and dehydrated for 1 week.

After dehydration, the delicate tissues of the specimens were cleared in 1% KHO solution for 2–3 days until the bones were clearly visible. The specimens were then transferred to a fresh solution of 1% KOH and were stained with a few drops of (0.4 ml) alizarine red. The staining continued overnight, and the over-staining was corrected by storing the specimens in Mall’s solution (79% distilled water, 20% glycerine, and 1% KOH). The specimens were then passed through increasing concentrations of glycerine (20%, 40% 60%, and 80%) for 1 week in each concentration. Finally, specimens were placed in 100% glycerine for evaluation. In addition, a small thymol crystal was added to prevent fungal growth and contamination during storage in pure glycerine.

Finally, each specimen was seen under a dissecting microscope with transparent background and bright-field optics. The degree of ossification of the sternebrae, metacarpal, metatarsal, and sacrococcygeal bones has been reported to be the primary indices of skeletal development in rats [66]. The extent and number of ossification centers in each bone of the fetuses were examined under a dissecting microscope. The skull, hyoid, sternebrae, ribs, vertebrae, and limb bones were carefully evaluated. Assessment of the skeletal development was performed by using a skeletal scoring chart, that was designed by Nash and Persaud [68]. After investigation, sample photomicrographs were taken with an automated built-in digital dissecting microscope camera (XTL3101, England) under 4× magnification.

#### Placental examination

From all groups, each placenta was examined for any gross morphological abnormalities. Furthermore, two- three Placentae/dam/groups were randomly selected for histopathologic examination. From each placenta, a sample in the size of 3 to 4 mm was taken, and it was fixed by dipping it in 10% formalin. Following an overnight fixation, the tissues were dehydrated by an ascending series of alcohol (40%, 50%, 70%, 80%, 90%, 100%). The tissues were then cleared by xylene (I, II, and III). After clearing, the tissues were impregnated with melted paraffin wax (I and II). Finally, each sampled tissue was placed in an embedding cassette and filled with melted wax. A 5 µm section was made for every block and the ribbon was placed on the frosted slide and then kept in a hot oven (40–45 °C) for 20–30 min [69]. Staining of the tissues was based on the following procedures: the slides were dewaxed with three steps of xylene for 5 min in each, rehydrated with descending series of alcohol (absolute alcohol I, absolute alcohol II, 90% alcohol, 80% alcohol, 70% alcohol) for 2 min in each, washed with running tap water for 2 min. Then the slides were stained with Harris hematoxylin for 5–10 min, cleaned with running tap water for 10 min, immersed in acid alcohol for 2–3 s, and counterstained with eosin for 1–2 min. The stained slides were dehydrated by ascending series of alcohol (80%, 95%, absolute alcohol I and II) for 2 min in each and cleared with xylene I, II, and III, 2 min in each. Finally, the cleared slides were mounted with DPX and covered with a coverslip [21]. In the stained slides, a senior pathologist investigated the structural integrity of the placenta using a binocular light microscope. The decidual zone, the labyrinthine zone, giant cells, and trophoblasts of the placenta were investigated, and important findings were photographed by an automated built-in digital microscope camera (Leica EC4, Germany) under 10× and 40× objective lens magnification.

### Statistical analysis

The data were coded, entered, and analyzed using Statistical Package for Social Sciences (SPSS) version 24. Statistical differences on fetal development indicators among all groups were analyzed by one-way analysis of variance (ANOVA) followed by a Tukey post hoc test for significant differences between the two groups. Furthermore, a chi-square test was performed to check if there was a difference in the percentages of organs and skeletal malformations between the treatment and control groups. The results are expressed as mean ± standard deviation of the mean (SDM) and percentages. Results with a *P-value* < 0.05 were considered statistically significant.

## Results

### Maternal food intake and weight gain

**Fig. 1:**
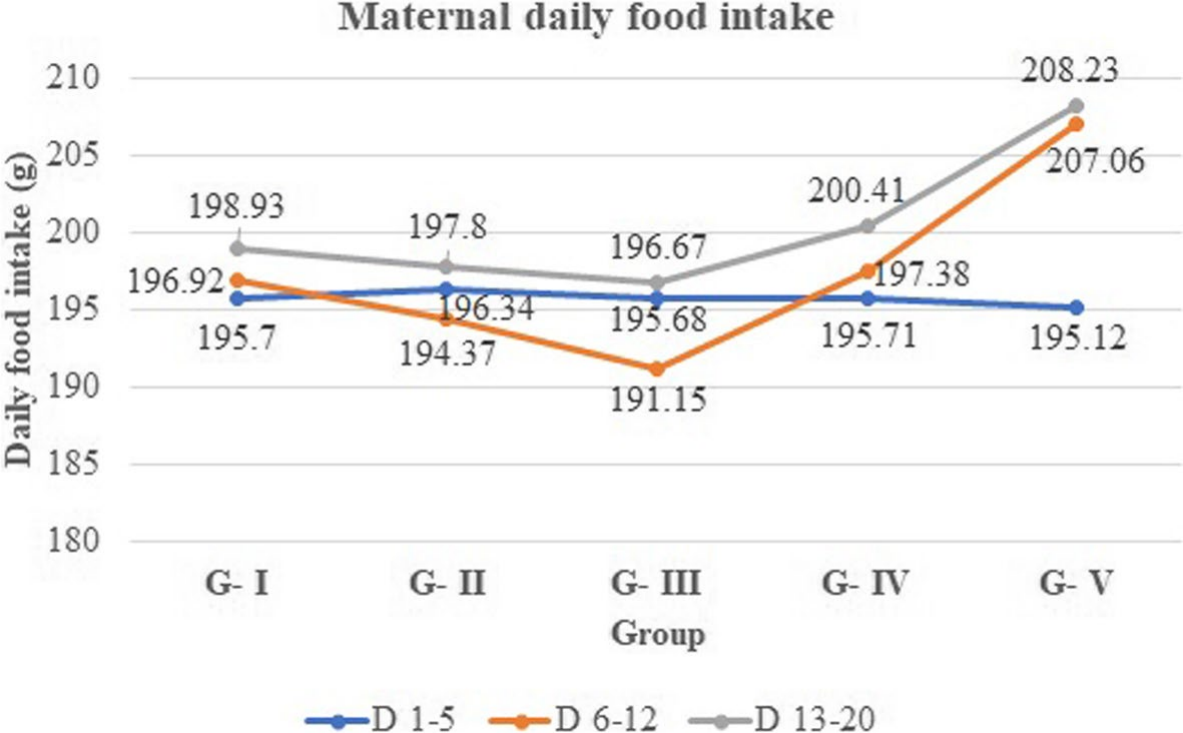
Mean maternal daily food intake (g/day) of pregnant rats treated with 70% ethanol leaf extract of* M. stenopetala*

In the current study, there was no significant difference in the maternal daily food intake during the pre-treatment period (days 1–5) between the treatment and control groups. However, the maternal daily food intake was reduced during the treatment (days 6–12) and the post-treatment periods (days 13–20) in all the treated groups compared with the pair-fed control group. However, it was not statistically significant. Figure 1 shows the maternal daily food intake of pregnant rats during the entire gestation period.

The maternal weight gain was determined during the days of the pregnancy before treatment (days 1–5), during treatment (days 6- 12), and after treatment (days 13– 20) periods. During the pre-treatment period, there was no significant difference in maternal weight gain between the treatment and control groups. However, it tended to decrease in the 1000 mg/kg treated group compared to the pair-fed control group, both during and after treatment periods. However, the difference was not statistically significant (Table 1).

**Table 1:** Maternal weight gain of pregnant rats following treatment with 70% ethanol *M. stenopetala* leaf extract

**Group**	**Maternal weight gain (in gram)**		
	**Day 1–5**	**Day 6 -12**	**Day 13–20**
**G-I: (250 mg/kg)**	12.2 ± 1.55	17.4 ± 3.81	62.8 ± 4.37
**G-II: (500 mg/kg)**	11.4 ± 2.12	17.1 ± 2.69	61.5 ± 3.14
**G-III: (1000 mg/kg)**	12.1 ± 1.60	16.3 ± 4.00	58.9 ± 10.00
**G-IV: (Pair fed control)**	11.8 ± 3.16	18.9 ± 2.51	65.1 ± 6.28
**G-V: (Ad libitum control)**	11.7 ± 1.70	19.0 ± 2.11	68.7 ± 4.95

Results are stated as mean ± standard deviation of mean; One Way ANOVA

### Pregnancy outcomes

**Fig. 2:**
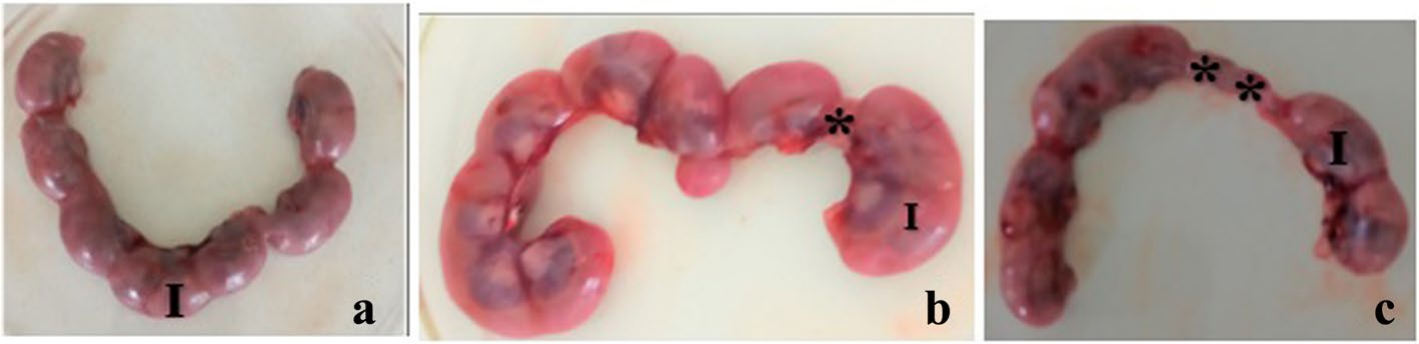
Showing implantation sites (I) and fetal resorption (*) on the gravid uterus of rat treated with 70% ethanol leaf extract of *M. stenopetala* leaf: **a** ad libitum control, **b** 500 mg/kg, **c** 1000 mg/kg groups

The pregnancy outcomes of the pregnant rats are summarized in Table 2 and shown in Fig. 2. The pregnancy outcomes of the rats in the present study were affected in the *M. stenopetala*-treated groups. In this study, treatment of pregnant rats with *M. stenopetala* leaf extract revealed a decrease in the number of implantation sites, the number of fetuses, and the number of live fetuses when compared with the pair-fed control group. However, it was not statistically significant. In addition, treatment of pregnant rats with 1000 mg/kg of *M. stenopetala* leaf extract showed a significant increase in fetal resorptions when compared with the low dose (250 mg/kg), middle dose (500 mg/kg) treated, and the pair-fed control groups (*p* < 0.05).

**Table 2:** Pregnancy outcomes of rats following treatment with 70% ethanol *M. stenopetala* leaf extract

**Variables**	**Treatment group**			**Control group**	
	**250 mg/kg**	**500 mg/kg**	**1000 mg/kg**	**Pair-fed control**	**Ad libitum control**
No. of fetuses	109	106	105	112	113
No. of implantation sites/litter	11.3 ± 0.48	11.0 ± 1.89	10.7 ± 0.82	11.5 ± 1.08	11.3 ± 1. 06
No. of resorption sites/litter	0.4 ± 0.70	0.4 ± 0.48	0.6 ± 0.42^a^	0.4 ± 0.48	0.4 ± 0.51
No. of live fetuses/litter	10.9 ± 0.74	10.6 ± 1.58	10.1 ± 0.53	11.2 ± 1.14	11.3 ± 1.06
No. of dead fetuses/litter	0	0	0	0	0
Weight of gravid uterus	68.7 ± 7.64	68.3 ± 10.63	69.3 ± 4.75	67.0 ± 10.17	65.4 ± .39
No. of male fetuses/dam	5.8 ± 1.14	5.3 ± 0.82	4.6 ± 1.08	4.9 ± 1.29	6.0 ± 0.74
No. of female fetuses/dam	4.9 ± 0.88	5.3 ± 1.16	5.9 ± 0.74	6.1 ± 1.45	5.2 ± 1.14

The results are summarized as mean ± standard deviation of mean

*n* Number of pregnant rats

^a^Significantly different from 250 mg/kg, 500 mg/kg treated and pair-fed control groups: One Way ANOVA

### Fetal growth indicators

In the present study, fetal growth was evaluated by measuring the weights of the gravid uterus, fetal membrane, live fetuses, placenta, and CRL. When compared with the pair-fed control group, the fetal and placental weights were significantly reduced in the 1000 mg/kg treated group. Furthermore, the CRL of the fetuses was significantly decreased in the high-dose treated group when compared with the pair-fed control group. The CRL of the fetuses in the high-dose and the pair-fed control groups was 4.9 ± 0.47 cm and 5.6 ± 0.36 cm, respectively (Table 3).

**Table 3:** Fetal growth following treatment of 70% ethanol leaf extract of *M. stenopetala* leaf

**Groups**	**Fetal growth indices**			
	**WP/fetus (g)**	**WFM (g)**	**CRL/fetus (cm)**	**FW/fetus(g)**
**G-I: (250 mg/kg)**	0.66 ± 0.03	0.3 ± 0.03	5.1 ± 0.54	4.7 ± 1.33
**G-II: (500 mg/kg)**	0.67 ± 0.10	0.3 ± 0.09	5.0 ± 0.46	4.7 ± 0.80
**G-III: (1000 mg/kg)**	0.61 ± 0.04^a^	0.2 ± 0.03	4.9 ± 0.47^a^	4.3 ± 0.82^a^
**G-IV: (Pair fed control)**	0.71 ± 0.03	0.2 ± 0.03	5.6 ± 0.36	5.3 ± 0.91
**G-V: (Ad libitum control)**	0.72 ± 0.05	0.2 ± 0.06	5.6 ± 0.71	5.4 ± 0.44

The results are summarized as mean ± standard deviation of mean

*P*-value < 0.05; One Way ANOVA

*WGU* Weight of gravid uterus, *WP* Weight of placenta, *WFM* Weight of fetal membrane, *CRL* Crown ramp length, *FW* Fetal weight

^a^Significantly different compared with pair-fed control group

### External morphological anomalies

**Fig. 3:**
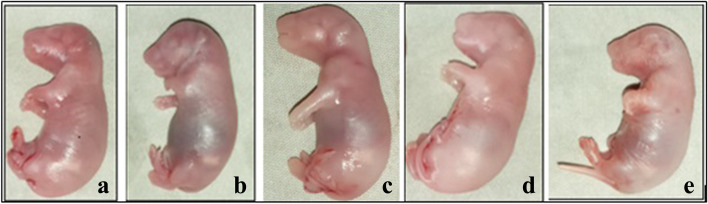
Shows live fetus from each group: **a** 250 mg/kg; **b** 500 mg/kg; **c** 1000 mg/kg; **d** pair-fed control and **e** ad libitum control groups

As shown in Fig. 3, visible abnormalities were not seen on the external body of the near-term rat fetuses in all the treatment and control groups.

### Visceral morphological anomalies

**Fig. 4:**
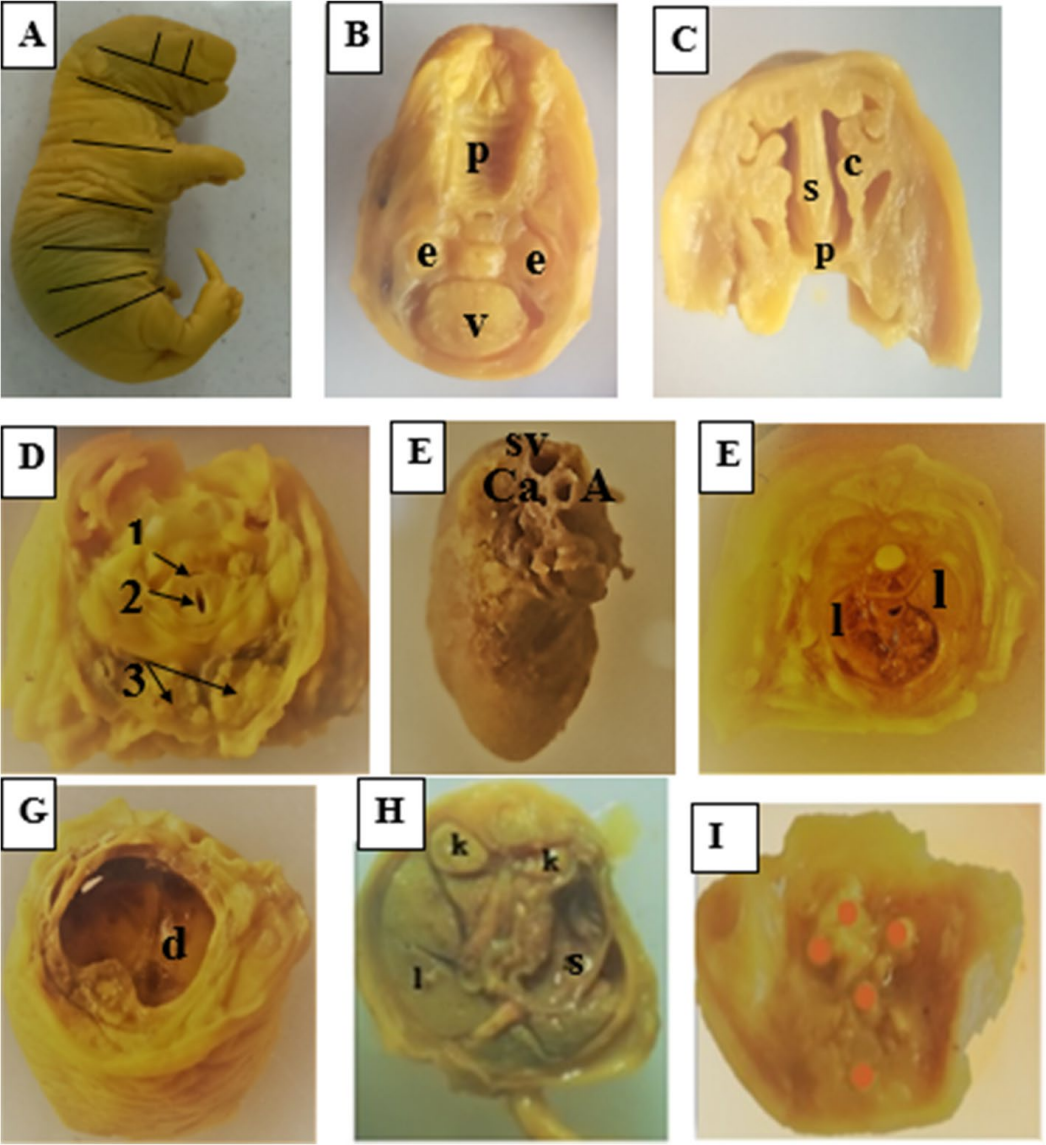
Bouin’s fixed fetuses for visceral examination (1000 mg/kg). **A** Un-sectioned fetus showing sites where sections made; **B** coronal section showing normal palate (p), eye ball (e) and brain ventricle; **C** coronal section of nasal cavity showing nasal septum (s), nasal conchae (c) and palate (p); **D** a section made through the neck showing normal 1-esophagus, 2-trachea, and 3-thyroid; **E** heart showing superior vena cava (SV), aorta (A), coronary artery (ca) **F** a section through the chest showing normal interventricular septum (s) and lungs (l) **G** intact diaphragm, **H** a section made through the abdomen showing normal visceral organs including the liver (l) kidney (k), stomach (s); **I** section showing pelvic visceral organs (doted)

In the current study, the visceral organs appeared normal in the treatment and the control groups. Developmental defects of the eyes, ventricles, nasal cavity, palate, oral cavity, thyroid, thymus, trachea, and esophagus were not observed. Similarly, no observable malformations in the development of the heart, lungs, diaphragm, abdominal visceral organs, and external genitalia in all the treatment and control groups (Table 4 and Fig. 4).

**Table 4:** Percentage of organ malformations in the fetal soft tissue following exposure of pregnant rats with 70% ethanol *M. stenopetala* leaf extract

**Groups**	**Percent of fetuses with malformed organ**								
	**Hc**	**E**	**Cp**	**H**	**L**	**K**	**FL**	**HL**	**Ti**
G-I: (250 mg/kg) (*n* = 80)	0	0	0	0	0	0	0	0	0
G-II: (500 mg/kg) (*n* = 80)	0	0	0	0	0	0	0	0	0
G-III: (1000 mg/kg) (*n* = 80)	0	0	0	0	0	0	0	0	0
G-IV: (Pair-fed control) (*n* = 80)	0	0	0	0	0	0	0	0	0
G-V: (Ad libitum control) (*n* = 80))	0	0	0	0	0	0	0	0	0

Results are expressed as percentage of fetuses with malformed organ (chi-square)

G-I: 250 mg/kg, G-II: 500 mg/kg, G-III: 1000 mg/kg treated groups; G-IV: pair-fed and G-V: ad libitum control groups

*Hc* Hydrocephalus, *E* Eyes, *Cp* Cleft palate, *H* Heart, *L* Liver, *K* Kidneys, *FL* Fore limbs, *HL* Hind limbs, *Ti* Tail, *n* Number of fetuses examined

### Skeletal malformations

**Fig. 5:**
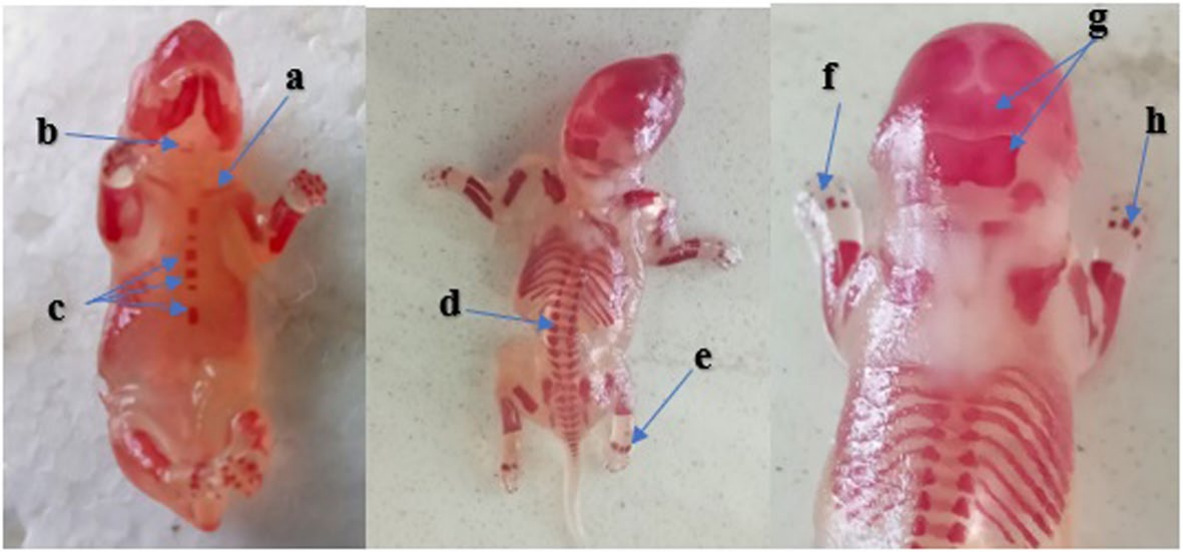
Alizarin red stained 20 days old rat fetuses showing different ossification centers. a: clavicle; b: hyoid; c: sternum; d: vertebrae; e: metatarsals; f: forelimb phalanges; g: supra-occipital and interparietal; h: metacarpals

The findings from skeletal evaluations are summarized in Tables 5 and 6 and Fig. 5. According to the observations, skull bones, thoracic vertebrae, ribs, and hyoid bone did not show any skeletal malformations in all the treatment and control groups. However, differences in the ossification centers were observed in the sternum, sacro-caudal vertebrae, metacarpus, metatarsus, forelimb phalanges, and hindlimb phalanges between the treatment and control groups. Yet, it was not statistically significant. When compared with the low-dose treated and the pair-fed control groups, however, a statistically higher percentage (40.7%) of rat fetuses had no proximal hindlimb phalanges in the high-dose treated group.

**Table 5:** Skeletal malformations of 20 days old rat fetuses following treatment of pregnant rats with 70% ethanol extract of *M. stenopetala* leaf

**Groups**	**Percent of skeletal malformations**						
	**Hyoid**^**a**^	**Sternum**^**a**^	**Rs**^**b**^	**CV**^**d**^	**TV**^**b**^	**LV**^**d**^	**SCV**^**c**^
**G-I: ((*n* = 27)**	0	11.1	0	0	0	0	7.4
**G-II (*n* = 27)**	0	14.8	0	0	0	0	11.1
**G-III (*n* = 27)**	0	22.2	0	0	0	0	18.5
**G-IV (*n* = 27)**	0	11.1	0	0	0	0	7.4
**G-V (*n* = 27)**	0	7.4	0	0	0	0	7.4

Results are expressed as percentage of skeletal malformations (chi-square)

G-I: 250 mg/kg, G-II: 500 mg/kg, G-III: 1000 mg/kg treated groups; G-IV: pair-fed and G-V: ad libitum control groups

*CV* Cervical vertebrae, *TV* Thoracic vertebrae, *LV* Lumbar vertebrae, *SCV* Sacro-caudal vertebrae

^a^Sternum with less than 4 ossification centers and hyoid bone not showing signs of ossification

^b^Thoracic vertebrae with less than 13 ossification centers and with less than 13 ribs

^c^Caudal vertebrae with less than 4 ossification centers

^d^Cervical vertebrae with less than 7 ossification centers and Lumbar vertebrae with less than 5 ossification centers

**Table 6:** Skeletal (limb bones) malformations of 20 days old rat fetuses following treatment of pregnant rats with 70% ethanol extract of *M. stenopetala* leaf

**Groups**	**Percent of skeletal malformations of limb bones**			
	**Metacarpus**^**a**^	**Forelimb phalanges**^**b**^	**Metatarsal**^**a**^	**Hindlimb phalanges**^**b**^
**G-I: ((*n* = 27)**	7.4	18.5	0	18.5
**G-II (*n* = 27)**	11.1	22.2	7.4	33.3
**G-III (*n* = 27)**	14.8	25.9	11.1	40.7*
**G-IV (*n* = 27)**	11.1	11.1	0	18.5
**G-V (*n* = 27)**	7.4	11.1	0	14.8

Results are presented as a percentage (%) of skeletal malformations (chi-square test)

^*^Significantly different compared with 250 mg/kg treated and pair-fed control groups (*P*-value < 0.05)

^a^Presence of ≤ 3 metacarpus and metatarsus

^b^Absent proximal phalanges

### Effects on the placenta of 20 days old rat fetuses

#### Gross examination of placenta

**Fig. 6:**
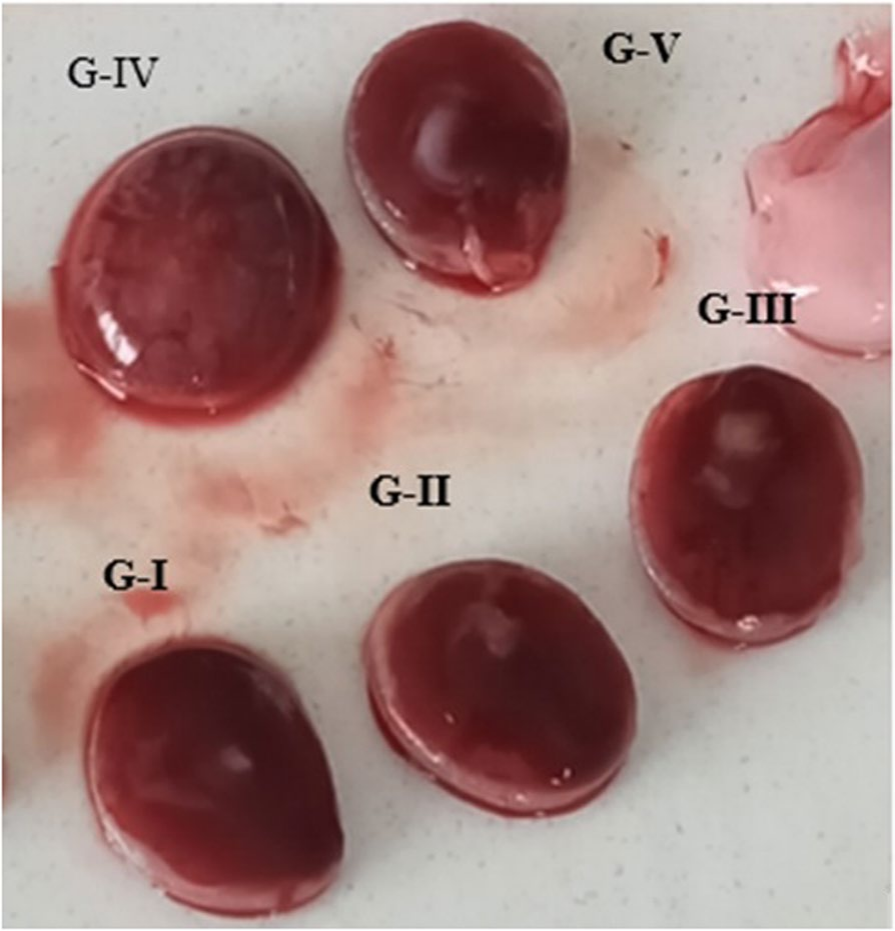
Shows sample of placentae taken from each group; G-I: 250 mg/kg; G-II: 500 mg/kg; G-III: 1000 mg/kg; G-IV: pair-fed control and G-V: ad libitum control groups

In the present study, the placentae were examined for the presence of any gross and microscopic changes. As shown in Fig. 6, there were no visible differences in the size, color, and gross appearance of the placentae between the treatment and control groups.

#### Examination of placental histopathology

**Fig. 7:**
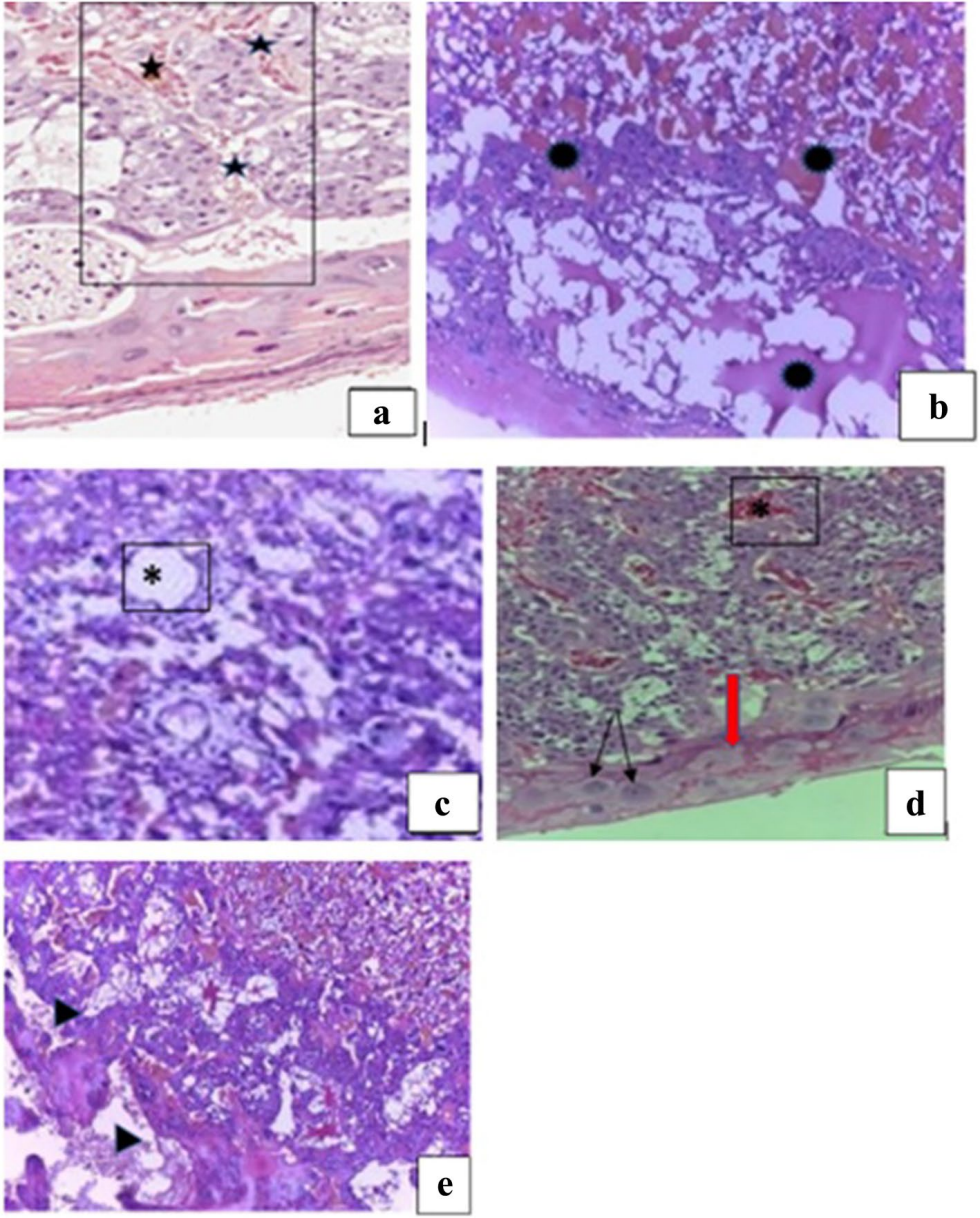
Photomicrographs of the placenta of rats treated with 1000 mg/kg/day of *M. stenopetala* leaf extracts showing; **a** trophoblast proliferation (  ); **b** hemorrhage in the trophoblastic and labyrinth zones (  ); **c** capillary dilatation (*); **d** decidual apoptosis (red arrow), decidual cytolysis (black arrows); **e** decidual necrosis (head arrow); H&E stain, **a** & **b** 100× and **c**, **d** & **e** 40× magnification

**Fig. 8:**
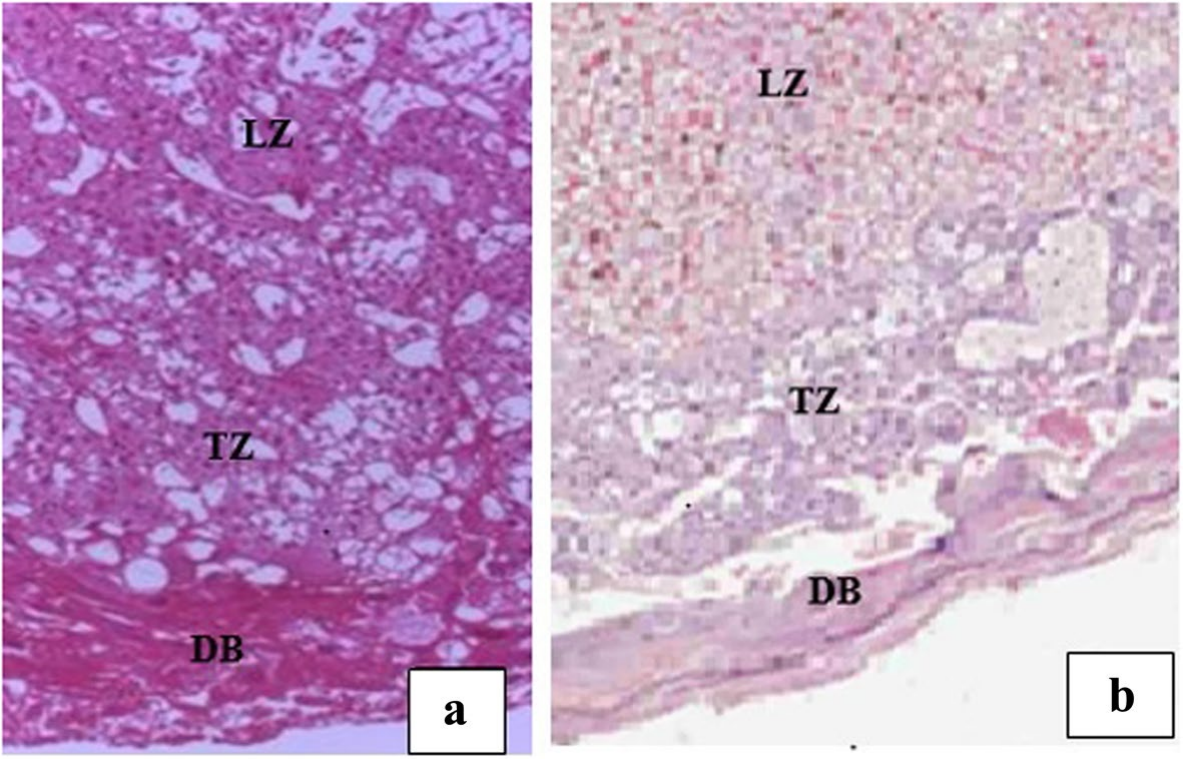
Photomicrographs of the placenta of pair-fed (**a**) and ad libitum (**b**) control groups of rats showing normal structural architecture: decidual basalis (DB); trophoblastic zone (TZ); and labyrinth zone (LZ); H&E stain, 40× magnification

Apart from the normal appearance of the gross structure of the placentae, however, light microscopic investigations revealed structural changes in the decidual basalis, trophoblastic, and labyrinthine zones of the placentae (Table 7). In the high-dose treated group, the trophoblastic and labyrinthine zones of the placentae were filled with hematoma (Fig. 7b). Similarly, capillary dilatation was seen in the placentae of all the treatment groups (Fig. 7c). Decidual necrosis was also evident in all the treatment groups (Fig. 7d). Likewise, decidual cytolysis and decidual apoptosis were observed in 500 mg/kg and 1000 mg/kg treated groups (Fig. 7e). Moreover, trophoblast proliferation was significantly increased in the high dose treated group (Fig. 7a) compared with the control groups (Fig. 8a & b). However, visible changes were not evident in the glycogen cells and spongiotrophoblast in all the treatment and control groups.

**Table 7:** Percentage of placental abnormalities of rats following exposure of pregnant rats with 70% ethanol *M. stenopetala* leaf extract

**Placental Abnormality (%)**	**Groups**				
	**G-I (*n* = 30)**	**G-II (*n* = 30)**	**G-III (*n* = 30)**	**G-IV (*n* = 30)**	**G-V (*n* = 30)**
Decidual degeneration	3.3	6.7	10	0	0
Decidual Apoptosis	0	3.3	13.3	3.3	0
Intervillous space thrombosis (hematoma)	0	0	3.3	0	0
Decidual Hypoplasia & Atrophy	0	0	6.7	0	0
Decidual Cytolysis	0	6.7	23.3	0	0
Capillary dilatation	3.3	6.7	13.3	0	0
Trophoblast proliferation	10	23.3	33.3*	16.7	13.3

Results are stated as percentage of placental abnormalities, Chi-square

G-I: 250 mg/kg; G-II: 500 mg/kg; G-III: 1000 mg/kg; G-IV: pair-fed control and G-V: ad libitum control groups

*n* Number of placentae examined

^*^Significantly different from ad libitum control group (*P*-value < 0.05)

## Discussion

In the current study, the in-vivo fetotoxicity of a 70% ethanol extract of *M. stenopetala* leaf was assessed. There were no deaths or conspicuous behavioural changes in the pregnant rats during the entire experiment between the treatment and control groups.

However, the maternal daily food intake and weight gain in the high dose treated group decreased during the treatment and post-treatment periods when compared with the pair-fed control group, but, not statistically significant. This finding agreed with another previous study that reported insignificant differences in maternal daily food intake and weight gain in mice treated with different doses of *M. stenopetala* leaf extracts [70]. But, the findings of our study were not consistent with the studies that revealed a significant reduction in maternal food intake and weight gain in rats treated with *M. stenopetala* extracts [57, 71]. This decrease in food consumption and the alterations in the animals’ body weight were constant, and it appears likely that toxicological factors are to blame [72]. The other possible explanation for weight changes in animals could be toxicity, disease progression, or a patient’s reaction to treatment [73, 74]. Moreover, the presence of tannins, one of the ingredients in *M. stenopetala* leaf, can harm the epithelial lining of the digestive tract and decrease nutrient absorption in rats, which in turn reduces food intake [74]. Another researcher revealed that rats given the same plant extracts saw alterations in body weight [50].

In addition, the possible reason for the decrease in the maternal daily food intake and weight gain in the pair-fed control group during the treatment and post-treatment periods when compared with the ad libitum control group might be due to the manipulation of animals during the experiment that may induce stress and leads to decrease in the daily food intake and weight gain. The other probable reason might be due to the restriction of animals from food. The rats were fed that was restricted and matched to the amount consumed by the experimental groups. However, rats in the ad libitum control group were unrestricted from food and water and they were not treated and not manipulated during the experiment. Consequently, manipulation of the rats during the experiment may induce stress and disturb the behaviour of the animals and affect maternal weight gain. This agreed with the study that reported an incidence of stress-induced weight loss in rats [75].

In the current study, the pregnancy outcomes of the rats that were treated with the high-dose of *M. stenopetala* leaf extract showed a significant difference from the pair-fed control group. The crown-rump length of a 20-day-old rat fetus was significantly decreased in the high dose treated pregnant rats. Similarly, a significant increase in the number of fetal resorptions was observed in the high dose treated pregnant rats. This agreed with another previous study that reported a higher incidence of fetal resorptions in pregnant rats treated with a high dose of methanolic extracts of *M. stenopetala* seeds [71]. This also agreed with another study that was conducted to investigate the abortion activity of *M. oleifera* in rats [76]. The possible justification for these analogous findings may be due to the similarities of the two plants that are grouped in the same plant family Moringaceae, as they may contain comparable active secondary metabolites that produce similar effects on the animals. Furthermore, these findings were consistent with another study that was conducted to investigate the teratogenic potential of high-dose *Syzygium guineense* leaves on rat embryos and fetuses [49]. The possible reason for producing similar findings from these two studies may be due to the existence of similar secondary metabolites like alkaloids in both plants that may have teratogenic potential on rat fetuses [40].

In the present study, possible indications of prenatal growth retardation were observed in near-term rat fetuses. The fetal and placental weights as well as the CRL were significantly reduced in the high-dose treated pregnant rats when compared with the pair-fed control group. Furthermore, a significant increase in the number of fetal resorptions was observed in the 1000 mg/kg treated pregnant rats. The reason for this developmental delay in fetal growth may be because of the active secondary metabolite; alkaloids that are present in the leaf of *M.* *stenopetala* that may cause disruption in the cholinergic neurotransmission and lead to developmental defects in the fetuses [38].

One aspect of development that occurs late in the gestation period is the mineralization of osseous tissue, and it is an indication of fetal maturity [77]. In this manner, the current study investigated the ossification status of 20 days old rat fetuses following exposure of pregnant rats to the test plant. The skull bones, thoracic vertebrae, ribs, and hyoid bone did not indicate any skeletal malformations in all the treatment and control groups. However, variations in the ossification centers were observed in the sternum, Sacro-caudal vertebrae, metacarpus, metatarsus, forelimb phalanges, and hindlimb phalanges between the treatment and control groups. So far, it has not been statistically significant. This suggested that ethanol extract of *M. stenopetala* leaf may not exert an adverse effect on the skeletal formation during fetal development in rats.

Placenta is a feto-maternal organ that exists temporarily during pregnancy. It plays a vital role in the local exchange of necessary nutrients, gases, wastes and immunoglobulins between the mother and the developing embryos and fetuses. It also provides flow of chemical information like drugs and toxins between the exposed mother and fetuses [78]. Consequently, it is the most susceptible target organ for direct chemical induced toxic insults and various placental toxic agents have been reported. In this way, histopathological examination of the placenta plays a critical role in the understanding of the mechanism of embryotoxicity and developmental toxicity and could benefit reproductive toxicity studies [79, 80].

In the present study, the placental weight was significantly reduced in the 1000 mg/kg treated rats. Furthermore, histopathological changes like trophoblastic proliferation, decidual degeneration, hematoma in the trophoblastic and labyrinthine zones, capillary dilation and decidual cellular apoptosis were observed in the treatment groups. Nevertheless, except the trophoblastic proliferation, none of the alterations were statistically significant. This is consistent with other previous studies conducted to investigate toxic effects of medicinal plants and reported treatment related reduction of placental weight and histopathological alterations [50, 74, 79]. The most likely reason for placental weight reduction and histological changes may be due to the bioactive components found in the tested plant; like terpenoids that can cross the placenta and may disturb the placenta and fetal growth [38]. Another explanation could be that the plant’s alkaloids and terpenoids have been connected to higher levels of bone morphogenetic protein-7 (BMP7), a kind of transforming growth factor (TGF), also known as osteogenic protein-1, in the decidua of the placenta, which is close to the site of implantation [41].

## Conclusion

In conclusion, feeding *M. stenopetala* leaves during gestation may be unsafe to developing fetuses. The plant’s toxic effects were seen in fetal developmental delays, which were shown by a decrease in the weights of the fetuses and placenta, as well as a fall in the fetuses CRL. As a result, it is advised to limit overfeeding *M. stenopetala* leaves during pregnancy. It is also recommended to undertake more research on the developmental statuses of animals besides rats while taking into account various prenatal developmental stages.

## Data Availability

All the necessary data used to support the results of this study are included in the manuscript.

## Abbreviations

AAU: Addis Ababa University
AAUMF: Addis Ababa University Medical Faculty
ANOVA: Analysis of variance
CRL: Crown-rump length
EPHI: Ethiopian Public Health Institute
IRB: Institutional Review Board
H&E: Haematoxyline and eosin
KOH: Potassium hydroxide
M: Moringa
OECD: Organization for Economic Cooperation and Development
SEM: Standard deviation of mean
SPSS: Statistical package for social sciences
TMMRD: Traditional and Modern Medicine Research Directorate
WHO: World Health Organization
